# DSE inhibits melanoma progression by regulating tumor immune cell infiltration and VCAN

**DOI:** 10.1038/s41420-023-01676-8

**Published:** 2023-10-13

**Authors:** Lin Xia, Maoxiao Feng, Yidan Ren, Xiaodong Hao, Qinlian Jiao, QinChen Xu, Yunshan Wang, Qin Wang, Ningji Gong

**Affiliations:** 1 Department of Plastic Surgery, Qilu Hospital, Cheeloo College of Medicine, Shandong University, Jinan, Shandong 250012 China; 2 Department of Clinical Laboratory, Shandong Provincial Hospital Affiliated to Shandong First Medical University, Jinan, 250021 Shandong China; 3 Department of Clinical Laboratory, The Second Hospital of Shandong University, 247 Beiyuan Street, Jinan, 250033 Shandong China; 4 Department of Anesthesiology, Qilu Hospital, Cheeloo College of Medicine, Shandong University, 107 Wenhua Xi Road, Jinan, 250012 Shandong China; 5 Department of Emergency, The Second Hospital, Cheeloo College of Medicine, Shandong University, Jinan, Shandong 250012 China

**Keywords:** Cancer, Tumour immunology, Oncogenes

## Abstract

Dermatan sulfate epimerase (DSE) is a C5 epiminase that plays a key role in converting chondroitin sulfate into dermal sulfate. DSE is often upregulated during carcinogenesis of some types of cancer and can regulate growth factor signaling in cancer cells. However, the expression and function of DSE in human melanoma have not been reported. In this study, we investigated the influence of tumor-derived DSE in melanoma progression and the potential mechanism of their action. First, proteomic analysis of collected melanoma tissues revealed that DSE was significantly down-regulated in melanoma tissues. DSE silenced or overexpressed melanoma cells were constructed to detect the effect of DSE on melanoma cells, and it was found that the up-regulation of DSE significantly inhibited the proliferation, migration and invasion of melanoma cells. Data analysis and flow cytometry were used to evaluate the immune subpopulations in tumors, and it was found that the high expression of DSE was closely related to the invasion of killer immune cells. Mechanistically, DSE promoted the expression of VCAN, which inhibited the biological activity of melanoma cells. Together, these results suggest that DSE is downregulated in melanoma tissues, and that high expression of DSE can promote melanoma progression by inducing immune cell infiltration and VCAN expression.

## Introduction

Melanoma is one of the most aggressive and dangerous types of cancer. According to a recent statistical analysis of data, the global incidence of melanoma exceeds 300,000 new cases diagnosed in 1 year, and its incidence has steadily increased over the past few decades, posing an even greater challenge to global health care systems [1–3]. The most common form of this cancer is cutaneous melanoma, which occurs on the skin. Influenced by an individual’s genetic background and ultraviolet radiation, random accumulation of somatic mutations or genetic defects causes normal melanocytes to become malignant melanomas [4, 5]. Over the years, the mortality rate associated with melanoma has increased with morbidity, reaching 1 in 4 deaths [6]. Melanoma initially appears as a radial growth stage lesion that spreads along the superficial layer of the epidermis. If diagnosed at this early stage, local lesions can be successfully removed with surgery. However, melanoma can rapidly transition to a vertical growth stage, in which it invades the dermis and then metastasizes to lymph and distant organs such as the lungs [7, 8], liver [9] and brain [10](stage III and IV, respectively), and these later stages are associated with a sharp decline in median 5-year survival [11]. Therefore, it is necessary to explore the molecular mechanisms that lead to melanoma development and metastasis, and to discover other therapeutic targets and biomarkers to improve patient survival.

Dermatan sulfate epimerase (DSE) is a C5 epiminase that plays a key role in converting chondroitin sulfate (CS) into dermal sulfate (DS). In some tissues, DSE increases structural diversity for CS/DS hybrid polysaccharide complexes [12]. The CS/DS chain is essential for the formation of its function by regulating the movement and activity of growth factors, proteases, cytokines, and chemokines. This characteristic of CS/DS leads to the regulation of various cellular behaviors during development [13]. Chondroitin sulfate (CS) is one of the main types of Glycosaminoglycan (GAG), and GAG is the main component of extracellular matrix of normal organs and tumor tissues. In tumors, GAG may not only be related to disease progression, but also specific glycan structures can be used as biomarkers and pharmacological targets for disease diagnosis [14–16]. Previous reports have shown that DSE is often upregulated during carcinogenesis in certain types of cancer, such as glioma and squamous cell carcinoma, and can regulate growth factor signaling in cancer cells [17, 18]. However, DSE mRNA and protein are frequently downregulated in hepatocellular carcinoma tumors and induce HCC malignant phenotypes by enhancing CCL5 [19]. These studies suggest the important role of DSE in tumorigenesis. However, its expression and function in human melanoma have not been reported.

The purpose of this study was to investigate the correlation between DSE expression and melanoma progression and the molecular mechanism that promotes melanoma progression. In this study, we found that the level of DSE in melanoma tissues was significantly reduced, and the high expression of DSE inhibited the growth, invasion and migration of melanoma cells. In terms of mechanism, DSE inhibits the progression of melanoma by regulating the intratumoral invasion of anti-tumor immune cells and the expression of Versican (VCAN). In conclusion, our study shows that the DSE-VCAN axis is significantly inhibited in melanoma tissues, and activation of this pathway is expected to be a new way of melanoma treatment.

## Results

### DSE is underexpressed in melanoma tissue

**Fig. 1:**
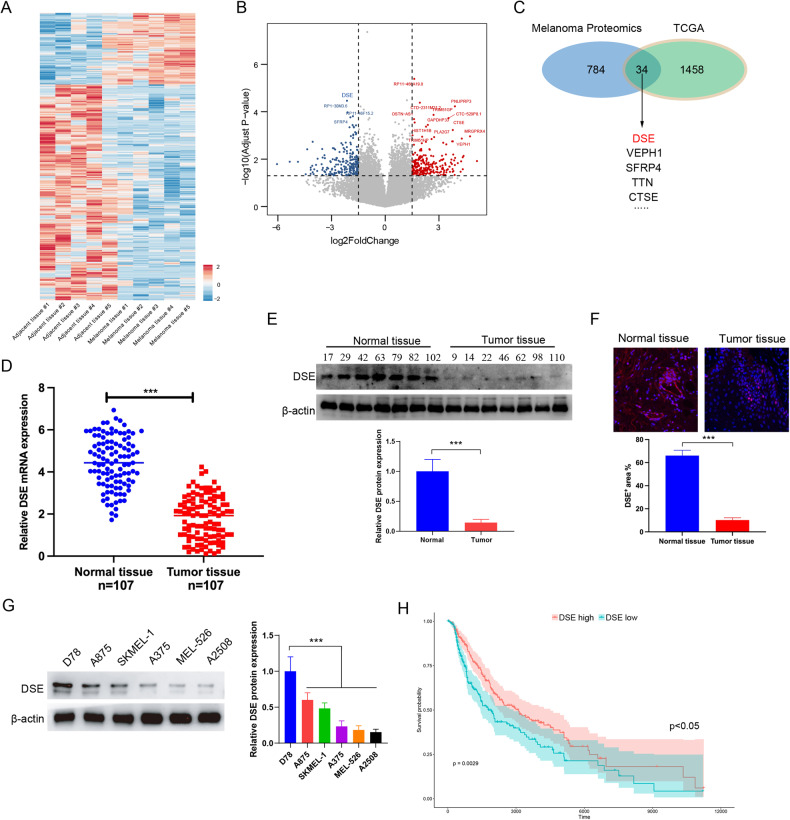
DSE is specifically low expressed in melanoma patients. **A** Proteomics was used to analyze human melanoma and paracancerous tissues, and heat maps showed proteins specifically expressed in human melanoma tissues; **B** Volcanic maps showed differentially up-expressed (291) and down-expressed (301) proteins; **C** Combined analysis of specific expression in human melanoma and differential genes in human melanoma in TCGA; **D** qRT PCR analysis of mRNA levels of DSE in human melanoma and normal tissues (mean ± s.e.m. ***P < 0.001); **E** Western blot analysis of DSE levels in human melanoma and normal tissues; **F** The expression of DSE in human melanoma and normal tissues was analyzed by fluorescence staining (mean ± s.e.m. ***P < 0.001); **G** Expression of DSE protein in human melanoma cell line; **H** TCGA data were used to analyze the effect of DSE expression level on the prognosis of patients with human melanoma. Statistical significance was assessed by two-tailed unpaired Student’s *t*-test (**D**–**G**). Data are representative of three (**D**–**G**) independent experiments.

In order to explore the important factors affecting the occurrence and development of melanoma, we collected 5 pairs of melanoma tissues and paracancer tissues for proteomic analysis. Sequencing analysis showed that 291 proteins were differentially upregulated and 301 proteins were significantly down-regulated in melanoma patients (Fig. 1A, B). Subsequently, we conducted a joint analysis of proteomic data and TCGA human melanoma RNA-seq data, and found that the mRNA and protein levels of 34 genes, including DSE, VEPH1, SFRP4, TTN, STSE, were consistently down-regulated in melanoma tissues (Fig. 1C). The downregulation of DSE was the most significant among the screened differentially expressed genes, so we selected DSE for subsequent study. To verify the low expression of DSE in melanoma tissues, 107 melanoma and 107 normal tissues were collected for qRT-PCR and Western blot. The results showed that the mRNA and protein levels of DSE were significantly down-regulated in melanoma tissues (Fig. 1D, E). Moreover, immunofluorescence staining further demonstrated that DSE was significantly underexpressed in melanoma tissues (Fig. 1F). In addition, we further examined the protein expression of DSE in a variety of melanoma cell lines, and found that DSE expression was decreased in most melanoma cell lines, while A875 cells maintained high DSE expression (Fig. 1G).

Further, the influence of DSE on melanoma was discussed. The TCGA database was used to analyze the influence of DSE expression level on the prognosis of melanoma patients, and Kaplan-Meier survival curve showed that patients with low DSE expression had poor prognosis (p < 0.01) (Fig. 1H). Together, these results suggest that DSE is significantly down-regulated in melanoma tissue and leads to poor prognosis in melanoma patients.

### DSE inhibits proliferation, invasion and migration of melanoma cells

**Fig. 2:**
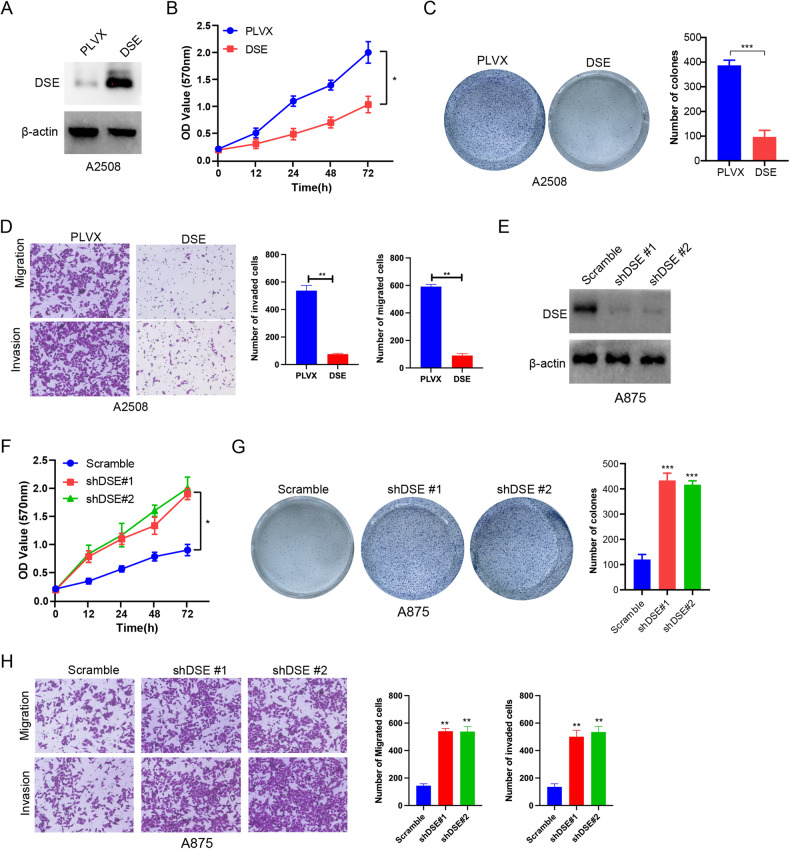
DSE inhibits proliferation, invasion and migration of melanoma cells. **A** A2508 cells were infected with lentivirus (PLVE) and DSE, respectively, and the expression levels of DSE protein in cells were analyzed by western blotting; **B** CCK8 assay to analysis cell viability of A2508 cells stably overexpressing DSE (mean ± s.e.m. *P < 0.05); **C** Colony formation assay to analyze cell proliferation of A2508 cells stably overexpressing DSE; (mean ± s.e.m. ***P < 0.001); **D** Transwell assay to analyze cell invasion and migration of A25O8 cells stably overexpressing DSE (mean ± s.e.m. **P < 0.01); **E** Knockdown of DSE in A875 cells and Western blot analysis of DSE protein expression levels in those cells; **F** CCK8 assay was used to analyze the cell viability of DSE knockout A875 cells; (mean ± s.e.m. **P < 0.01); **G** Colony formation assay to analyze cell proliferation of DSE knockout A875 cells (mean ± s.e.m. ***P < 0.001); **H** Transwell assay to analyze cell invasion and migration of DSE knockout A875 cells (mean ± s.e.m. **P < 0.01). Data are representative of three (**A**–**H**) independent experiments.

In order to reveal the function of DSE in melanoma, adenovirus transfection technique was used to construct melanoma cell lines with stable high expression or knockdown expression of DSE. Firstly, melanoma cells A2508 with relatively low DSE expression were selected and transfected with adenovirus with DSE overexpression. The efficiency of DSE overexpression was verified by Western blot (Fig. 2A and SFig 1A). The effect of DSE on the viability of melanoma cells was detected by CCK-8 assay, and it was found that overexpression of DSE significantly inhibited the proliferation of A2508 cells (Fig. 2B). In addition, consistent results were observed in MEL-526 cell lines with high DSE expression (SFig 1B, C). Colony formation experiments further demonstrated the inhibitory effect of DSE on melanoma cell proliferation (Fig. 2C). In addition, Transwell experiments showed that DSE overexpression significantly inhibited the invasion and migration ability of A2508 cells (Fig. 2D).

Subsequently, we selected the melanoma cell line A875 with relatively high DSE expression and knocked down its DSE expression by adenovirus transfection. The knockdown efficiency was shown in Fig. 2E and SFig. 1D. DSE knockdown significantly promoted the proliferation of melanoma cells A875 and SKMEL-1 through CCK-8 and/or colony formation assay (Fig. 2F, G and SFig. 1E, F). Moreover, DSE knockdown significantly inhibited the invasion and migration of A875 cells (Fig. 2H). Together, these in vitro results suggest that DSE inhibits proliferation, invasion, and migration of melanoma cells.

### DSE inhibits melanoma development and metastasis

**Fig. 3:**
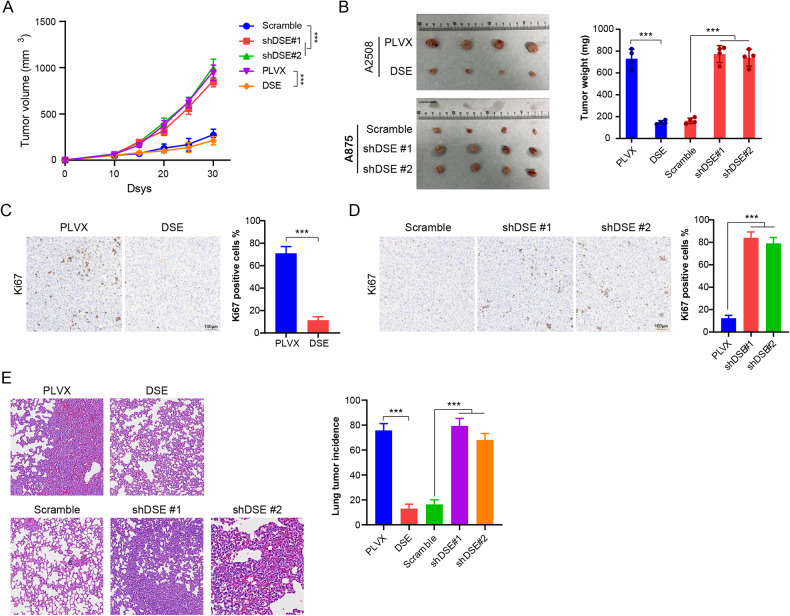
DSE inhibits melanoma growth and metastasis in vivo. **A** Analysis of the effect of DSE on tumor growth using cells overexpressing or knocking out DSE injected subcutaneously into nude mice (*n* = 4 mice, mean ± s.e.m. **P < 0.01); **B** Weight of tumor tissue in A (*n* = 4 mice, mean ± s.e.m. *P < 0.05, **P < 0.01); **C**, **D** Expression levels of Ki67 in tumor tissue in A (*n* = 4 mice, mean ± s.e.m. ***P < 0.001); **E** Tail vein injection of cells overexpressing or knocking out DSE into nude mice to analyze lung metastasis (*n* = 4 mice, mean ± s.e.m. **P < 0.01).

To further verify these results in vivo, we constructed a mouse subcutaneous tumor model using DSE overexpressed or knockdown melanoma cells. As shown in the figure, the melanoma growth rate of A2508 cells overexpressing DSE was significantly lower than that of control A2508 cells. The tumor growth rate of A875 with low DSE was significantly higher than that of control A875 (Fig. 3A, B). Immunohistochemical analysis of Ki-67 expression in tumors showed that overexpression of DSE significantly inhibited Ki-67 expression in tumor tissues, while knockdown of DSE significantly promoted Ki-67 expression (Fig. 3C, D). These results suggest that DSE significantly inhibits the development of melanoma in vivo.

In addition, we constructed a tumor lung metastasis model by injecting melanoma cells into the tail vein of nude mice, and found that DSE overexpression significantly inhibited melanoma lung metastasis, while DSE knockdown promoted melanoma metastasis (Fig. 3E).

### DSE enhances anti-tumor immunity

**Fig. 4:**
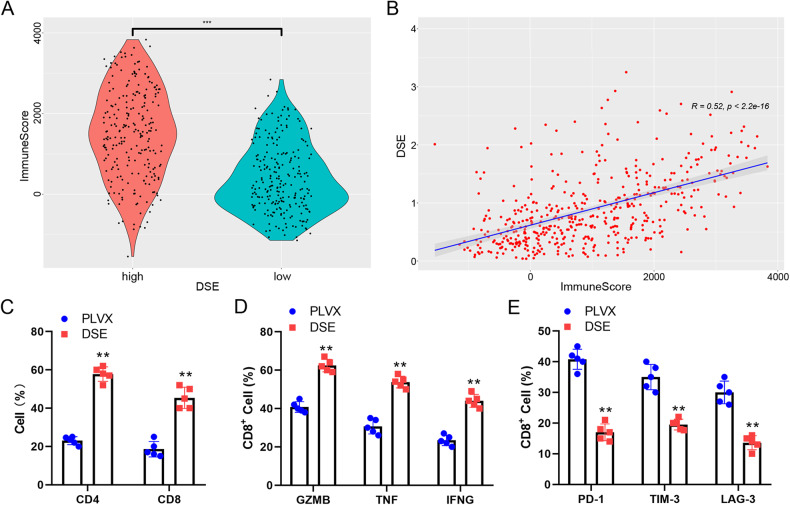
DSE enhances the antitumor immune microenvironment. **A** Analysis of immune infiltration scores in TCGA human melanoma patients; **B** Correlation analysis of immune infiltration scores with DSE expression levels; **C** Subcutaneous inoculation of B16 cells into C57/B6 mice and collection of lymphocytes infiltrating within the tumor for flow analysis of CD4^+^ and CD8^+^ T cell ratios (*n* = 5 mice, mean ± s.e.m. **P < 0.01); **D** Flow analysis of GZMB in CD8 + T cells, TNF and IFNG expression levels in CD8^+^ T cells (*n* = 5 mice, mean ± s.e.m. **P < 0.01); **E** Flow analysis of PD-1, TIM-3 and LAG-3 expression levels in CD8^+^ T cells (*n* = 5 mice, mean ± s.e.m. **P < 0.01).

Given the strong ability of DSE to inhibit tumor growth, we speculate that it may be closely related to the activation of anti-tumor immunity. An immunoinfiltration analysis based on TCGA melanoma cohort data showed that DSE was highly positively correlated with the immunoinfiltration score of tumor tissue (Fig. 4A, B). In addition, DSE was significantly positively correlated with stroma score (SFig 2A, B). CIBERSORT analysis further found that high DSE expression was positively correlated with infiltration of CD8^+^ T, activated CD4^+^ memory T cells and M1 macrophages, and negatively correlated with infiltration of M2 type macrophages (SFig 2C, D).

To further verify the results, we established a mouse subcutaneous tumor model using B16 melanoma cells and analyzed it by flow cytometry. Melanoma that overexpressed DSE showed slower tumor growth and lower tumor weight (SFig 3). Moreover, overexpression of DSE significantly promoted the invasion of CD4+ and CD8 + T cells into the tumor (Fig. 4C). In addition, overexpression of DSE promoted the overexpression of tumor killer molecules such as GZMB, TNF and IFNG in CD8 + T cells, and inhibited the expression of inhibitory molecules such as PD-1, TIM-3 and LAG-3 (Fig. 4D, E).

**Fig. 5:**
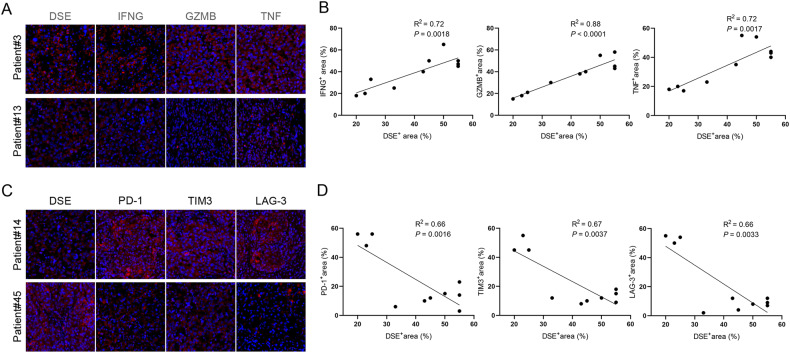
DSE in melanoma patient tissues is proportional to antitumor immune effector molecules. **A** Immunofluorescence staining of DSE, IFNG, GZMB and TNF in melanoma patient tissue; **B** Statistical analysis of the correlation between DSE and IFNG, GZMB and TNF expression levels; **C** Immunofluorescence staining of DSE, PD-1 in melanoma patient tissue, TIM3 and LAG3; **D** Statistical analysis of the correlation between DSE and PD-1, TIM3 and LAG3 expression levels.

In addition, tumor tissues from melanoma patients were collected for immunofluorescence staining. Tumor tissues with high DSE expression showed higher levels of IFNG, GZMB, and TNF expression (Fig. 5A, B). However, the expression of DSE was negatively correlated with the expression of PD-1, TIM3 and LAG3 (Fig. 5C, D). These results suggest that DSE inhibits the progression of melanoma by promoting the infiltration of immune cells and the expression of immunoeffector molecules.

### DSE promotes the expression of VCAN

**Fig. 6:**
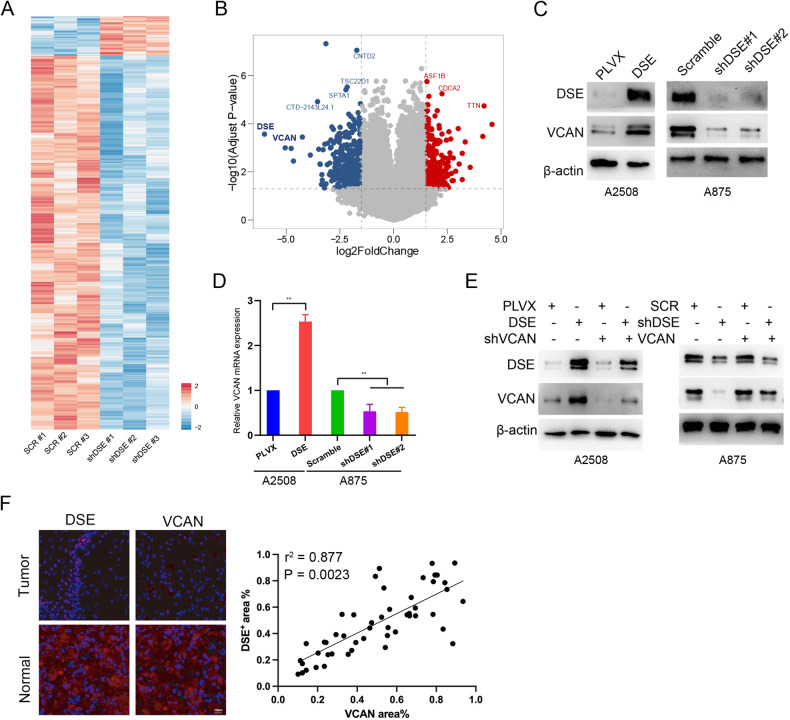
DSE regulates the expression of VCAN. **A** Transcriptome analysis of DSE-knockdown A875 cells; **B** Volcano plot showing differentially expressed genes in DSE-knockdown A875 cells; **C**–**E** VCAN protein and mRNA expression in DSE-overexpressing or knockdown cells level (mean ± s.e.m. **P < 0.01); **F** The correlation between DSE and VCAN expression in melanoma tissues was analyzed by immunofluorescence. Data are representative of three (**C**–**E**) independent experiments.

In order to further explore the molecular mechanism by which DSE inhibits melanoma progression, the transcriptome of control A875 and A875 cells with knockdown DSE was analyzed. Differential gene analysis showed that VCAN was most significantly down-regulated in DSE knockdown cell lines (Fig. 6A, B). Consistently, we found that VCAN protein levels in DSE overexpressed A2508 cells were significantly increased compared to control A2508 cells, while VCAN protein levels in DSE knockdown A875 cells were significantly decreased compared to control A875 cells by Western blot analysis (Fig. 6C, E and SFig 4). qRT-PCR also obtained relatively consistent results (Fig. 6D).

The correlation between DSE and VCAN expression in melanoma tissues was analyzed by immunofluorescence staining, and a significant positive correlation was found between DSE expression and VCAN expression (Fig. 6F). These results suggest that DSE promotes the expression of VCAN in melanoma tissues, which may be related to the inhibitory effect of DSE on melanoma.

### DSE regulates melanoma proliferation, invasion and migration through VCAN

**Fig. 7:**
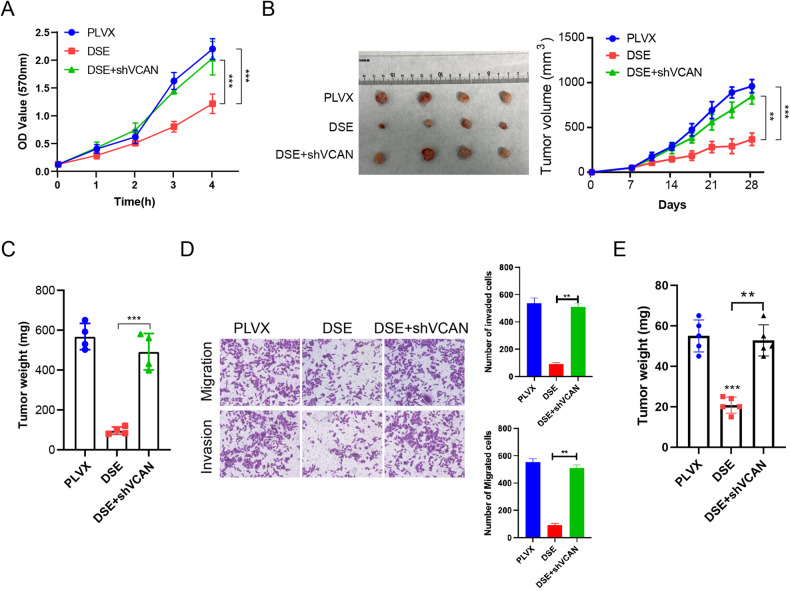
DSE regulates melanoma proliferation, invasion and migration through VCAN. **A** CCK8 assay to analyze the cell viability of A2508 cells overexpressing DSE and knocking out VCAN (mean ± s.e.m. **P < 0.01); **B** A2508 cells overexpressing DSE and knocking out VCAN were injected into nude mice to observe tumor growth; (*n* = 4 mice, mean ± s.e.m. **P < 0.01 and ***P < 0.001); **C** tumor weight in B (*n* = 4 mice, mean ± s.e.m. **P < 0.01)**; D** Transwell assay analyzing DSE overexpressing and VCAN knockout Invasion and migration of A2508 cells (mean ± s.e.m. **P < 0.01); **E** Tail vein injection of cells overexpressing or knocking out DSE into nude mice to analyze lung metastasis (*n* = 4 mice, mean ± s.e.m. **P < 0.01 and ***P < 0.001). Data are representative of three (**A**–**D**) independent experiments.

To verify the regulatory role of the DSE-VCAN axis in melanoma progression, A2508 cells with DSE overexpression and VCAN knockdown were selected for subsequent experiments. DSE overexpression significantly inhibited the proliferation of melanoma cells, while VCAN knockdown inhibited the effect of DSE overexpression (Fig. 7A). Further, the above melanoma cells were injected subcutively into nude mice to observe tumor growth. Tumor volume and weight measurements showed that DSE overexpression significantly inhibited melanoma growth, while VCAN knockdown reversed the inhibitory effect of DSE on melanoma (Fig. 7B, C). Moreover, VCAN knockdown also significantly eliminated the inhibitory effect of DSE on melanoma invasion and migration (Fig. 7D). In addition, by injecting melanoma cells into the caudal vein of nude mice to construct a tumor lung metastasis model, we also found that VCAN knockdown eliminated DSE-inhibited melanoma lung metastasis (Fig. 7E). These results suggest that the effect of DSE on melanoma is reversed by knockdown VCAN, suggesting that DSE regulates melanoma growth, invasion and migration by regulating the expression of VCAN.

## Discussion

Melanoma research has made significant progress in the last 10 years, with 13 new melanoma treatments including targeted therapies and immunotherapies approved in the United States [20]. Currently, common therapies for melanoma include targeted inhibitors of BRAFV600E and MEK kinases in the mitogen activated protein kinase (MAPK) pathway, interleukin-2 (IL-2), oncolytic viruses, interferon, and emerging immune checkpoint inhibitors (ICI) alone or in combination. The objective response rate was 61% in patients treated with the combination of anti-ctLA-4 and anti-PD-1, and the 5-year survival rate was 52% in patients treated with the combination [21–24]. Despite such great progress, current treatments do not cure most patients with metastatic melanoma. Metastatic melanoma is a refractory genetic disease, particularly 50% of tumors associated with the wild-type BRAF [25]. To make matters worse, many patients have primary or acquired resistance to existing treatments. Most patients with BRAF mutations usually initially show a response to BRAF inhibitors, but melanoma patients with BRAF mutations usually relapse only a few months after starting treatment [26]. In addition, most patients do not respond or have severe adverse reactions to immunotherapy, which is also very limited [27]. Given these melanoma treatment dilemmas, there is a need to explore novel molecular mechanisms that contribute to the development of melanoma metastases and to discover other therapeutic targets and biomarkers to improve patient survival.

Remodeling of extracellular matrix (ECM) in the tumor microenvironment modulates tumor progression. Chondroitin sulfate (CS) proteoglycans occur on ECMs and cell surfaces and can be catalyzed by DSE to form chondroitin sulfate/dermal sulfate (CS/DS) hybrid chains that mediate multiple growth factor signaling [18]. Initially, DSE was highly expressed in squamous cell carcinoma of different origins, and therefore was considered as a cancer cell antigen, named squamous cell carcinoma antigen recognized by T-cells 2 (SART2) [28]. However, in this study, the proteomics of melanoma tissues combined with the RNA-seq analysis of the melanoma group of TCGA found that the mRNA and protein levels of DSE in melanoma tissues were significantly decreased compared with paracancer tissues. Our studies on collected melanoma tissues and cells also found that DSE protein was consistently underexpressed in melanoma tissues. In addition, low expression of DSE promotes melanoma cell growth, invasion and metastasis, and is closely associated with poor prognosis of melanoma patients.

The relationship between DSE and tumor immunity is rarely reported. However, it has been found in previous studies that both CS and DS show affinity for cytokines and chemokines [29, 30]. These include CCL5, which plays an important role in mediating immune cell transport and activity [31, 32]. Therefore, DSE expression in melanoma may induce infiltration of CCl5-producing killer T cells and natural killer cells. Both data analysis and our animal models suggest that high expression of DSE in melanoma induces intratumoral infiltration of immune cells and promotes intratumoral levels of killer molecules. In addition, our results suggest that DSE regulates the progression of melanoma by regulating the expression of VCAN. Versican is one of the extracellular matrix (ECM) proteoglycans that are increased during inflammatory processes in many diseases such as cardiovascular disease, autoimmune diseases, and several cancers [33–35]. Versican interacts with versican assembly molecules in the matrix to form a stable scaffold for inflammatory cells. Versican also further affects inflammation by interacting with a variety of growth factors and cytokines involved in regulating inflammation, thereby affecting its bioavailability and bioactivity [36, 37]. Versican has been reported to be expressed in a wide range of malignancies such as ovarian [38], hepatocellular [39], colon [40], and bladder cancers [41] and is associated with poor patient outcomes. Versican has been associated with several classic features of cancer, such as proliferative signaling, avoidance of growth inhibition signals, resistance to cell death, angiogenesis, and tissue invasion and metastasis. However, we provide evidence that VCAN may perform the opposite function in melanoma cells. Knockdown of VCAN expression in DSE-overexpressed melanoma A2508 cells eliminated the tumor suppressive effect of DSE.

In conclusion, our results suggest that DSE levels in melanoma tissues are significantly reduced, and that high expression of DSE inhibits the biological activity of melanoma cells. In terms of mechanism, DSE inhibits the progression of melanoma by regulating the intratumoral invasion of anti-tumor immune cells and the expression of VCAN. Therefore, activation of the DSE-VCAN axis in melanoma tissue is expected to be a new way of melanoma treatment.

## Methods

### Melanoma data collection

RNA sequencing and clinical characteristics data of 472 tissue from the TCGA-SKCM dataset (https://xenabrowser.net/datapages/) are extracted. Melanoma and adjacent tissues were obtained from the Second Hospital of Shandong University for proteomic analysis. Tissue collection has obtained informed consent from each patient. All experiments were approved by the Research Ethics Committee of the Second Hospital of Shandong University (approval number: KYLL-2022-312).

### Cell culture

The cell lines used in this study were all from Chinese Academy of Sciences Cell Bank (Shanghai, China). D78 cell, A375 cell, MEL526 and A2058 were cultured by Dulbecco’s Modified Eagle’s Medium (DMEM) culture (including 10% FBS) at 37 °C in the culture box with 5% CO2. A875 cell and SKMEL-2 cell were cultured by MEM (including NEAA) culture (including 10% FBS) at 37 °C in the culture box with 5% CO2. The culture medium was changed after 48 h. The subculture was digested when the degree of fusion had reached 80%.

### Constructs and transfections

Stable knockdown of DSE or VCAN in melanoma cell lines was generated by adenovirus-based shRNA delivery. Specific target shRNAs were subcloned into lentiviral vector pADM-U6-shRNA-mCMV-copGFP (Ji-Nan Weizhen), and a non-target shRNA was used as a negative control.

### Quantitative real-time PCR (RT-qPCR)

Total RNA was isolated from cells or tissues using the animal tissue RNA extraction kit and subjected to cDNA synthesis using BeyoRT™ II cDNA First Strand Synthesis Kit according to the manufacturer’s instructions. Quantitative real-time PCR was performed with cDNA samples mixed with 2×SYBR Green Premix Taq on a Step One Plus Real Time PCR system (Applied Biosystems, QuantStudio 6 Flex). The mRNA expression level of the gene to be tested was obtained by calculating 2^(-△△Ct).

### Western blot

Cells were washed in ice-cold PBS and solubilized in lysis buffer [25 mM Tris-HCl pH 7.6, 200 mM NaCl, 1 mM EDTA, 1% Igepal CA-630, 0.1% Na-Deoxycholate, 0.1% SDS supplemented with a mixture of protease inhibitors]. The lysates were incubated on ice for 30 min and centrifuged at 12,000 rpm for 25 min. All supernatants were collected and quantified for protein concentration using BCA reagent (Pierce). The lysates were subjected to SDS-PAEG gel electrophoresis, transferred to PVDF membrane and blocked with 5% BSA following manufacturer’s instructions. The membranes were incubated with primary antibodies at 4 °C overnight. After extensive washing and incubation with the corresponding secondary antibodies, protein bands were developed and captured with the imaging system (UVITEC, Alliance Q9 Advanced Auto). DSE antibodies were purchased from Abcam (PA5-100488), and VCAN antibodies were purchased from Invitrogen (MA5-27638).

### Immunofluorescence staining

Tissue samples were fixed with formalin and processed to paraffin blocks. Six-micrometer sections were cut, mounted on slides, and dried, then deparaffinized in xylene and ethanol. For antigen retrieval, slides were boiled for 20 min in 10 mM sodium citrate buffer (pH 6.0) and cooled for 30 min at room temperature. Sections were incubated overnight at 4 °C for triple immunofluorescence with a mixture of primary antibodies in a humid chamber. Then, sections were rinsed for 10 min in PBS and incubated for 1 h at room temperature with a mixture of secondary antibodies in a humid chamber. Sections were mounted in VECTASHILED (Vector Laboratories Inc., Burlingame, CA) mounting medium, and confocal microscopy images were acquired (LSM 780, Zeiss). Image J software was used to analyze the area proportion of positive signals in tissue immunofluorescence. DSE antibody were purchased from SIGMA (HPA014764), VCAN antibody were purchased from Invitrogen (MA5-27638).

### Cell proliferation and colony formation assay

Cells were seeded (3000 cells/well) and cultured in 96-well plate for 24, 48, 72 and 96 h. At each time point, 20 µL of prepared CCK-8 solution (Beyotime) was added for OD570 measurement with Thermo ScientificTM VarioskanTM LUX (Thermo Scientific). For colony formation assay, 700 cells were inoculated in 6-well plates and cultured for 10 days. The cells were then immobilized with 4% paraformaldehyde for 15 min. Finally, dye with 0.5% crystal violet for 15 min. Then Image J software (National Institutes of Health, Bethesda, MD, USA) was used to count the colonies. Import the image into the software, set the color and parameters. Finally, the software counts the specific colonies.

### Cell migration and invasion assay

In the migration assay, 1 × 10^4^ cells were seeded into the upper chamber of Transwell (8.0 μm pore size; Corning) with 200 µL of serum-free medium. The lower chambers contained 800 µL medium supplemented with 20% FBS. In the invasion assay, the upper chambers were coated with matrigel (Corning) and seeded with 1 × 10^4^ cells, and the lower chambers were filled with 800 µL 20% FBS medium. After incubation at 37˚C, 5% CO2 for 48 h, the Transwell chambers were taken out and the medium in the well was discarded, and then washed with PBS. The cells were then fixed with methanol for 30 min and stained with 0.1% crystal violet for 20 min. The upper unmigrated cells were gently wiped off with a cotton swab before counting under microscope. Three independent assays were performed.

### TME Cell Infiltration analysis

CIBERSORT algorithm was used to quantify the proportion of tumor-infiltrating immune cells based on the LM22 signature from Cibersort, and the Gene signatures of 28 tumor-infiltrating lymphocytes (TILs) downloaded from TISIDB, respectively. Tumor purity scoring was performed by the “ESTIMATE” package in R.

### Melanoma mouse model

C57BL/6 mice (6–8 weeks old) were inoculated subcutaneously with control and DSE overexpression or knockdown melanoma cells (1 × 10^6^). Tumor size was measured as length×(width^2^)/2. Survival was recorded each day. Tumor-bearing mice were euthanized when tumor size exceeded 2000 mm^3^. For lung metastasis mouse model, the mice were randomly assigned to different experimental groups. Wild-type or treated (1×10^6^ suspended in PBS) melanoma cells were injected into the tail vein of mice. Twenty-one days after the tumor cells were injected, the lung was dissected to observe the metastases, and the fixed tissue sections were stained. All experiments were approved by the Research Ethics Committee of the Second Hospital of Shandong University (approval number: KYLL-2022-312).

### RNA-seq

Control or DSE knockdown A875 cells were cultured and total RNA was extracted from them. RNA-seq libraries were generated using NEBNext Ultra RNA Library Prep Kit for Illumina (NEB) and sequenced on an Illumina platform with 125 bp/150 bp paired-end reads. Clean data were obtained by removing reads containing adapter and poly-N as well as low-quality reads from raw data. Clean reads were mapped with the reference genome Hisat2 (version 2.0.5) based on the gene model annotation file. Feature Counts v1.5.0-p3 was used to count the read numbers mapped to each gene. Fragments per kilobase per million mapped reads of each gene were calculated based on the length of the gene and the read count mapped to this gene. Differential analysis of gene expression was performed by using the “limma” package in R with |log2FC | > 0.8 and adjusted *p*-value < 0.05.

### 4D label-free quantitative proteome analysis

Tumor and paracancer tissues were collected for proteomic analysis. The tissue samples were ground and transferred to a centrifuge tube, where four volumes of a cracking buffer containing a mixture of 8 M urea and 1% protease inhibitor were added, and then treated three times with a high-intensity ultrasonic processor (Scientz). Then centrifuge at 12,000 g at 4 °C for 10 min. After the residual debris was removed, the supernatant was collected and the protein concentration of the product was determined using the Bisincionic acid assay (BCA) kit. Trypsin was added to the treated protein solution at a ratio of 1:50 (trypsin to protein mass ratio), the first digestion was performed overnight, and the second digestion was performed at a ratio of 1:100 (trypsin to protein mass ratio) for 4 h. After solubilization by liquid chromatography, mobile phase A (aqueous solution containing 0.1% formic acid and 2% acetonitrile) was separated by NanoElute ultra-high performance liquid chromatography system. The peptides isolated by UHPLC system were ionized by capillary ion source and then analyzed by timsTOF Pro mass spectrometer. The non-standard quantization method adopts the LFQ quantization principle, and obtains the relative quantization value of each sample from the LFQ intensity, and corrects it through the retrieval database software.

### Flow cytometry

MagniSort Positive Selection Beads (eBioscience) were used for isolation of CD8^+^ and CD4^+^ Tcells following the manufacturer’s instructions. Cells were stimulated with Phorbol 12-Myristate 13-Acetate (PMA, 100 ng/ml) and ionomycin (1 μM) for 6 h and collected for flow cytometry analysis. Flow cytometry was performed on the FACS Aria Sorp system (BD FAC Aria II Sorp System). Flowjo 10.4 software was used to analyze the data.

### Statistical analysis

The data were presented as the means ± standard error of mean (SEM) of at least three independent experiments. Statistical differences of means were assessed by using unpaired Student’s t-test (two-tailed). *P*-values <0.05 (*P* < 0.05) were considered as statistically significant.

### Supplementary information

Original Data File

Supplementary Material

## Data Availability

All data generated and analyzed in this study are included in this published article and its supplementary information file. For more data, please contact the corresponding author.

## References

[1] Cress RD, Holly EA (1997). Incidence of cutaneous melanoma among non-Hispanic whites, Hispanics, Asians, and blacks: an analysis of California cancer registry data, 1988−93. Cancer Causes Control.

[2] Brandt MG, Moore CC (2019). Nonmelanoma skin cancer. Facial Plast. Surg. Clin. North Am.

[3] Ferlay J, Colombet M, Soerjomataram I, Parkin DM, Pineros M, Znaor A (2021). Cancer statistics for the year 2020: an overview. Int. J. Cancer.

[4] Harrison SL, MacLennan R, Speare R, Wronski I (1994). Sun exposure and melanocytic naevi in young Australian children. Lancet.

[5] Alexandrov LB, Nik-Zainal S, Wedge DC, Aparicio SA, Behjati S, Biankin AV (2013). Signatures of mutational processes in human cancer. Nature.

[6] Teixido C, Castillo P, Martinez-Vila C, Arance A, Alos L (2021). Molecular markers and targets in melanoma. Cells.

[7] Harpole DH, Johnson CM, Wolfe WG, George SL, Seigler HF (1992). Analysis of 945 cases of pulmonary metastatic melanoma. J. Thorac. Cardiovasc. Surg..

[8] Damsky WE, Rosenbaum LE, Bosenberg M (2010). Decoding melanoma metastasis. Cancers.

[9] Olofsson Bagge R, Nelson A, Shafazand A, All-Eriksson C, Cahlin C, Elander N (2023). Isolated hepatic perfusion with melphalan for patients with isolated uveal melanoma liver metastases: a multicenter, randomized, open-label, phase III trial (the SCANDIUM Trial). J. Clin. Oncol.

[10] Kleffman K, Levinson G, Rose IVL, Blumenberg LM, Shadaloey SAA, Dhabaria A (2022). Melanoma-secreted amyloid beta suppresses neuroinflammation and promotes brain metastasis. Cancer Discov.

[11] Centeno PP, Pavet V, Marais R (2023). The journey from melanocytes to melanoma. Nat. Rev. Cancer.

[12] Tykesson E, Hassinen A, Zielinska K, Thelin MA, Frati G, Ellervik U (2018). Dermatan sulfate epimerase 1 and dermatan 4-O-sulfotransferase 1 form complexes that generate long epimerized 4-O-sulfated blocks. J. Biol. Chem.

[13] Mizumoto S, Yamada S, Sugahara K (2014). Human genetic disorders and knockout mice deficient in glycosaminoglycan. Biomed. Res. Int.

[14] Jia XL, Li SY, Dang SS, Cheng YA, Zhang X, Wang WJ (2012). Increased expression of chondroitin sulphate proteoglycans in rat hepatocellular carcinoma tissues. World J. Gastroenterol.

[15] Lv H, Yu G, Sun L, Zhang Z, Zhao X, Chai W (2007). Elevate level of glycosaminoglycans and altered sulfation pattern of chondroitin sulfate are associated with differentiation status and histological type of human primary hepatic carcinoma. Oncology..

[16] Liu CH, Lan CT, Chou JF, Tseng TJ, Liao WC (2017). CHSY1 promotes aggressive phenotypes of hepatocellular carcinoma cells via activation of the hedgehog signaling pathway. Cancer Lett.

[17] Thelin MA, Svensson KJ, Shi X, Bagher M, Axelsson J, Isinger-Ekstrand A (2012). Dermatan sulfate is involved in the tumorigenic properties of esophagus squamous cell carcinoma. Cancer Res.

[18] Liao WC, Liao CK, Tsai YH, Tseng TJ, Chuang LC, Lan CT (2018). DSE promotes aggressive glioma cell phenotypes by enhancing HB-EGF/ErbB signaling. PLoS One.

[19] Liao WC, Yen HR, Liao CK, Tseng TJ, Lan CT, Liu CH (2019). DSE regulates the malignant characters of hepatocellular carcinoma cells by modulating CCL5/CCR1 axis. Am. J. Cancer Res.

[20] Li L, Fukunaga-Kalabis M, Herlyn M (2011). The three-dimensional human skin reconstruct model: a tool to study normal skin and melanoma progression. J. Vis. Exp.

[21] Eggermont AMM, Blank CU, Mandala M, Long GV, Atkinson V, Dalle S (2018). Adjuvant pembrolizumab versus placebo in resected stage III melanoma. N. Engl. J. Med.

[22] Eggermont AMM, Blank CU, Mandala M, Long GV, Atkinson VG, Dalle S (2021). Adjuvant pembrolizumab versus placebo in resected stage III melanoma (EORTC 1325-MG/KEYNOTE-054): distant metastasis-free survival results from a double-blind, randomised, controlled, phase 3 trial. Lancet Oncol.

[23] Bottomley A, Coens C, Mierzynska J, Blank CU, Mandala M, Long GV (2021). Adjuvant pembrolizumab versus placebo in resected stage III melanoma (EORTC 1325-MG/KEYNOTE-054): health-related quality-of-life results from a double-blind, randomised, controlled, phase 3 trial. Lancet Oncol.

[24] Robert C, Grob JJ, Stroyakovskiy D, Karaszewska B, Hauschild A, Levchenko E (2019). Five-year outcomes with Dabrafenib plus Trametinib in metastatic melanoma. N. Engl J Med.

[25] Klemen ND, Wang M, Rubinstein JC, Olino K, Clune J, Ariyan S (2020). Survival after checkpoint inhibitors for metastatic acral, mucosal and uveal melanoma. J. Immunother. Cancer.

[26] Ito T, Tanaka Y, Murata M, Kaku-Ito Y, Furue K, Furue M (2021). BRAF heterogeneity in melanoma. Curr. Treat Opt. Oncol.

[27] Eddy K, Chen S (2020). Overcoming immune evasion in melanoma. Int. J. Mol. Sci.

[28] Nakao M, Shichijo S, Imaizumi T, Inoue Y, Matsunaga K, Yamada A (2000). Identification of a gene coding for a new squamous cell carcinoma antigen recognized by the CTL. J. Immunol.

[29] Mizumoto S, Fongmoon D, Sugahara K (2013). Interaction of chondroitin sulfate and dermatan sulfate from various biological sources with heparin-binding growth factors and cytokines. Glycoconj. J.

[30] Martin L, Blanpain C, Garnier P, Wittamer V, Parmentier M, Vita C (2001). Structural and functional analysis of the RANTES-glycosaminoglycans interactions. Biochem..

[31] Halama N, Zoernig I, Berthel A, Kahlert C, Klupp F, Suarez-Carmona M (2016). Tumoral immune cell exploitation in colorectal cancer metastases can be targeted effectively by anti-CCR5 therapy in cancer patients. Cancer Cell.

[32] Sutton A, Friand V, Papy-Garcia D, Dagouassat M, Martin L, Vassy R (2007). Glycosaminoglycans and their synthetic mimetics inhibit RANTES-induced migration and invasion of human hepatoma cells. Mol. Cancer Ther.

[33] Gill S, Wight TN, Frevert CW (2010). Proteoglycans: key regulators of pulmonary inflammation and the innate immune response to lung infection. Anat. Rec..

[34] Hanahan D, Weinberg RA (2011). Hallmarks of cancer: the next generation. Cell.

[35] Sorokin L (2010). The impact of the extracellular matrix on inflammation. Nat. Rev. Immunol.

[36] Wight TN, Kang I, Merrilees MJ (2014). Versican and the control of inflammation. Matrix Biol.

[37] Frevert CW, Felgenhauer J, Wygrecka M, Nastase MV, Schaefer L (2018). Danger-associated molecular patterns derived from the extracellular matrix provide temporal control of innate immunity. J. Histochem. Cytochem.

[38] Li D, Wang X, Wu JL, Quan WQ, Ma L, Yang F (2013). Tumor-produced versican V1 enhances hCAP18/LL-37 expression in macrophages through activation of TLR2 and vitamin D3 signaling to promote ovarian cancer progression in vitro. PLoS One..

[39] Xia L, Huang W, Tian D, Zhang L, Qi X, Chen Z (2014). Forkhead box Q1 promotes hepatocellular carcinoma metastasis by transactivating ZEB2 and VersicanV1 expression. Hepatology..

[40] Bogels M, Braster R, Nijland PG, Gul N, van de Luijtgaarden W, Fijneman RJ (2012). Carcinoma origin dictates differential skewing of monocyte function. Oncoimmunol..

[41] Said N, Theodorescu D (2012). RhoGDI2 suppresses bladder cancer metastasis via reduction of inflammation in the tumor microenvironment. Oncoimmunol..

