# Supplementary figures and images for: DSE inhibits melanoma progression by regulating tumor immune cell infiltration and VCAN
Source: Cell Death Discov. 2023 Oct 13;9:373. doi: 10.1038/s41420-023-01676-8 (PMC10576081; doi:10.1038/s41420-023-01676-8)

Fig 1E


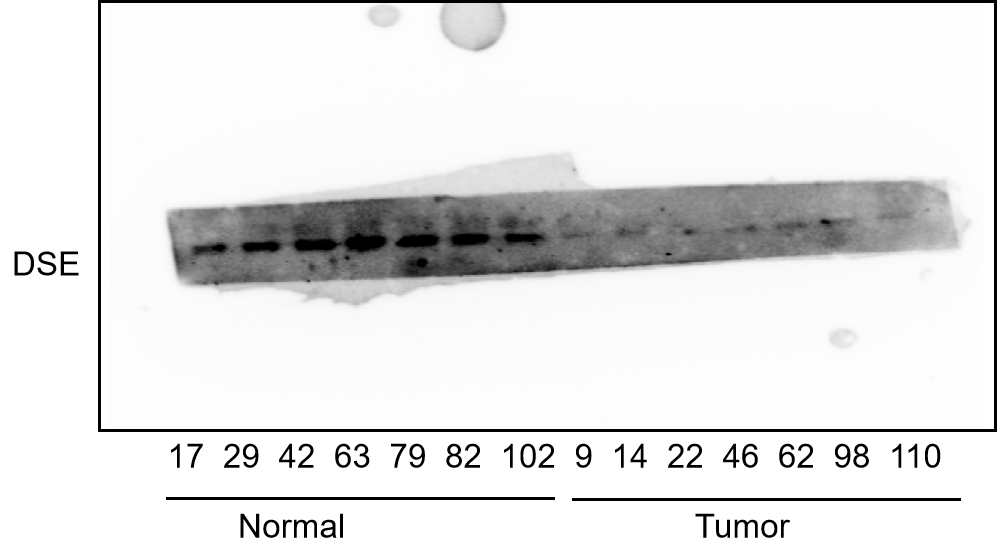


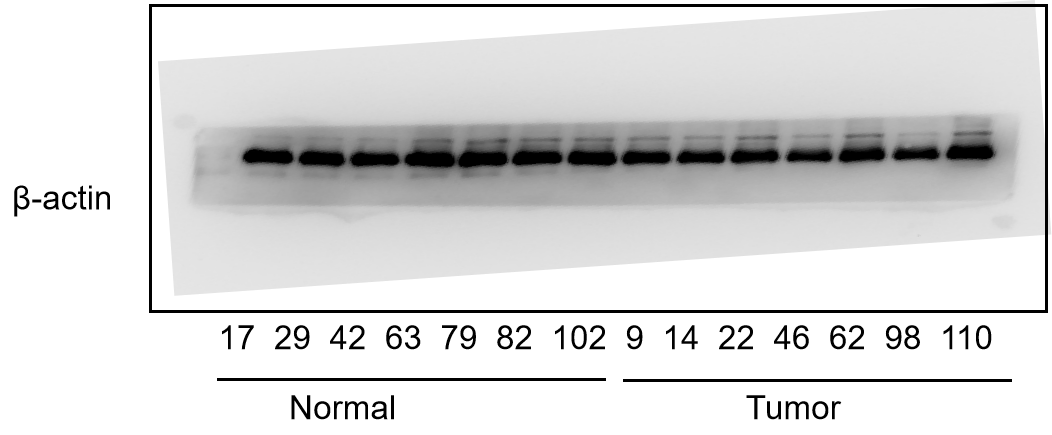


Fig 1G


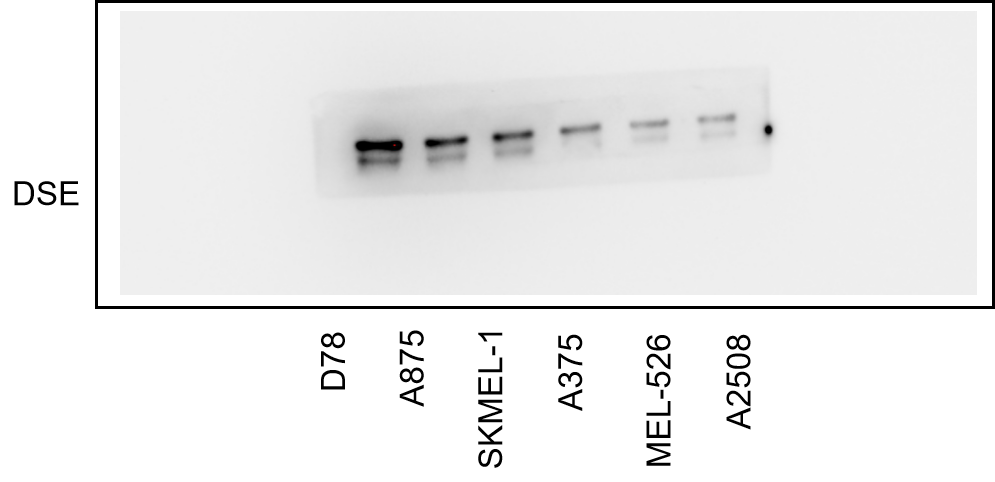


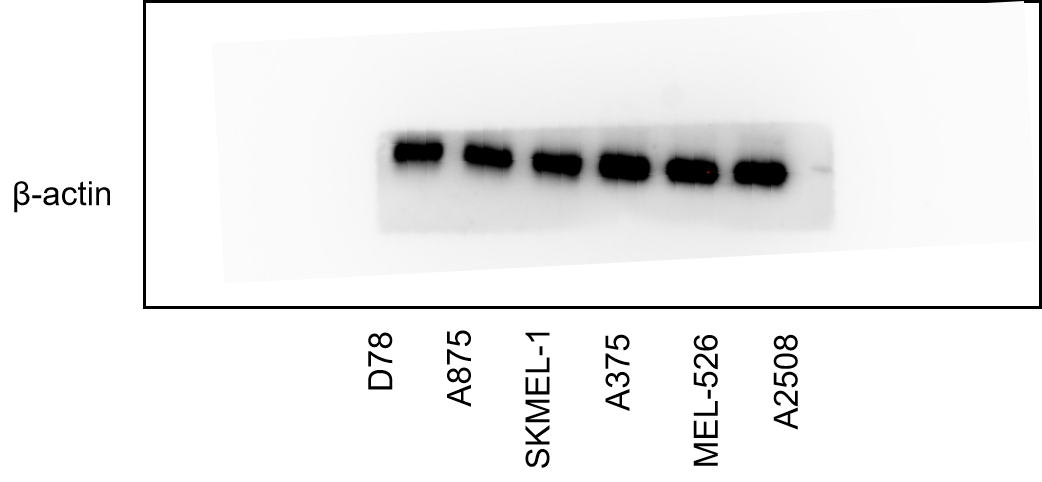


Fig 2A


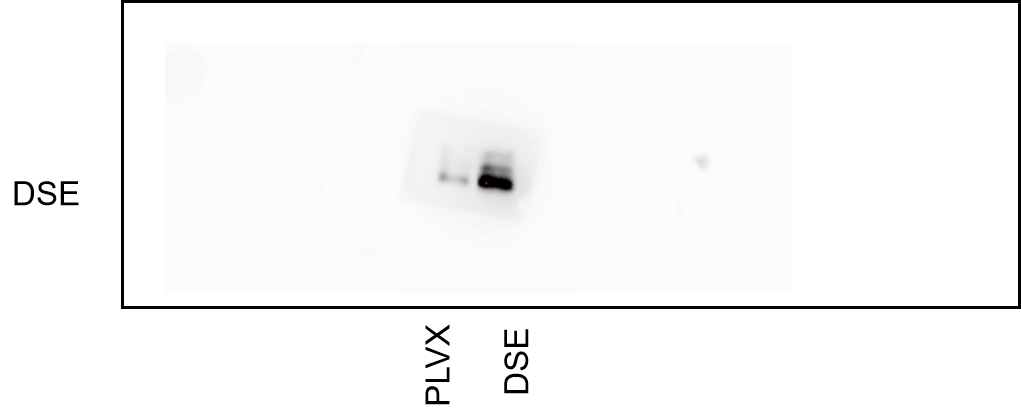


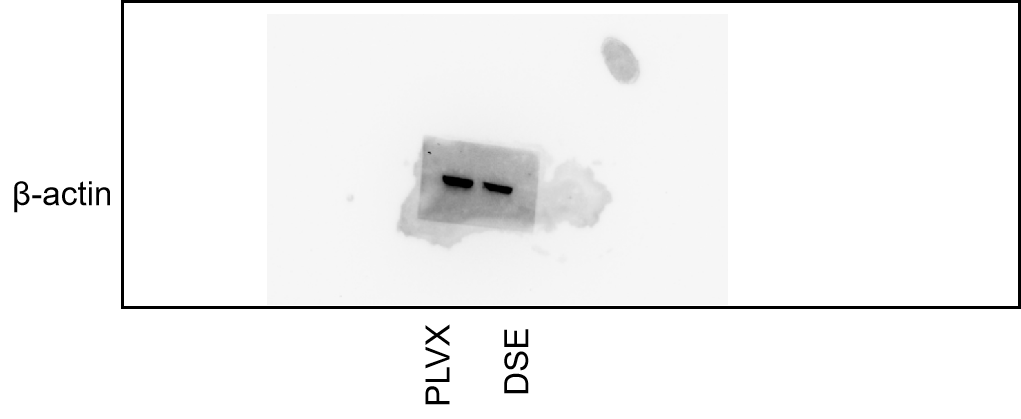


Fig 2E


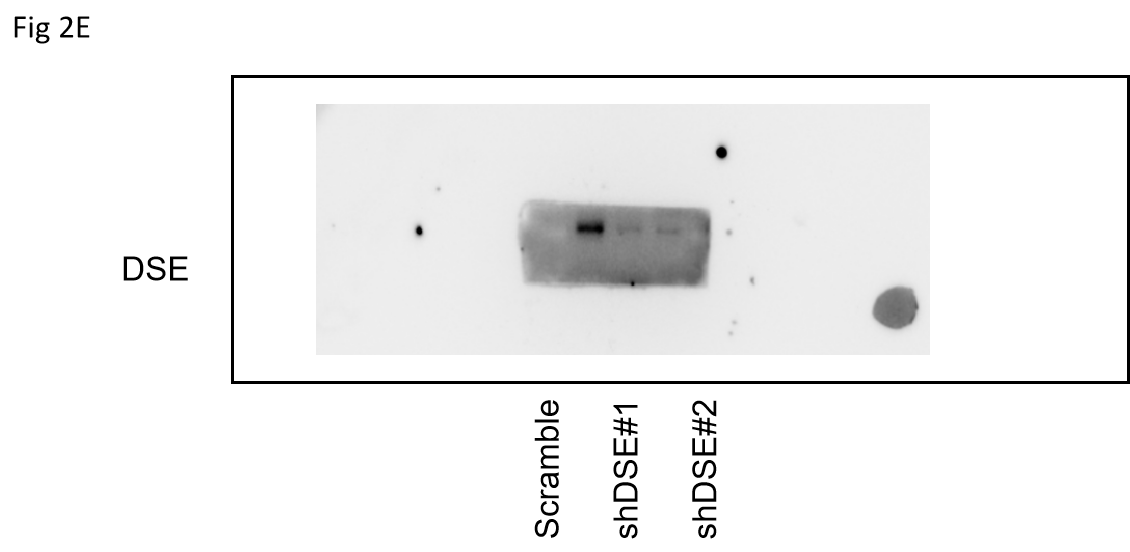


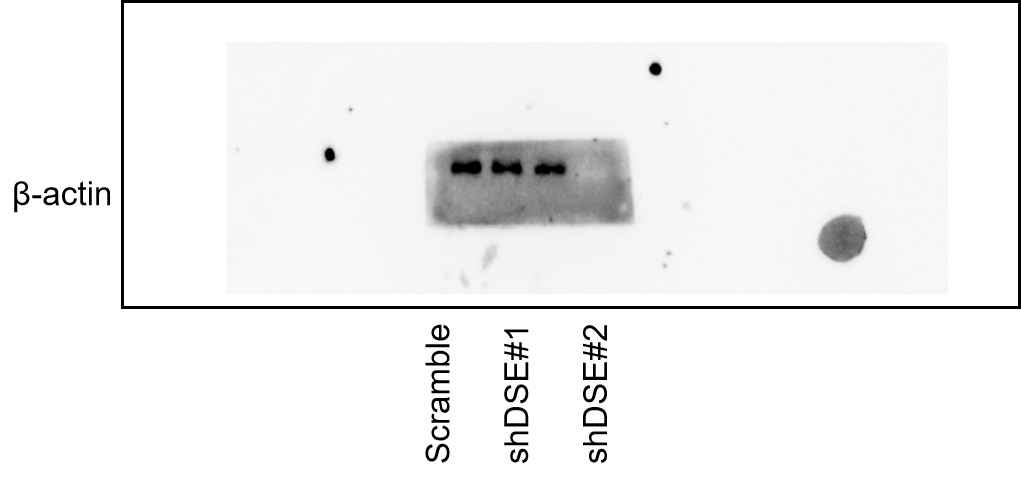


Fig 6C


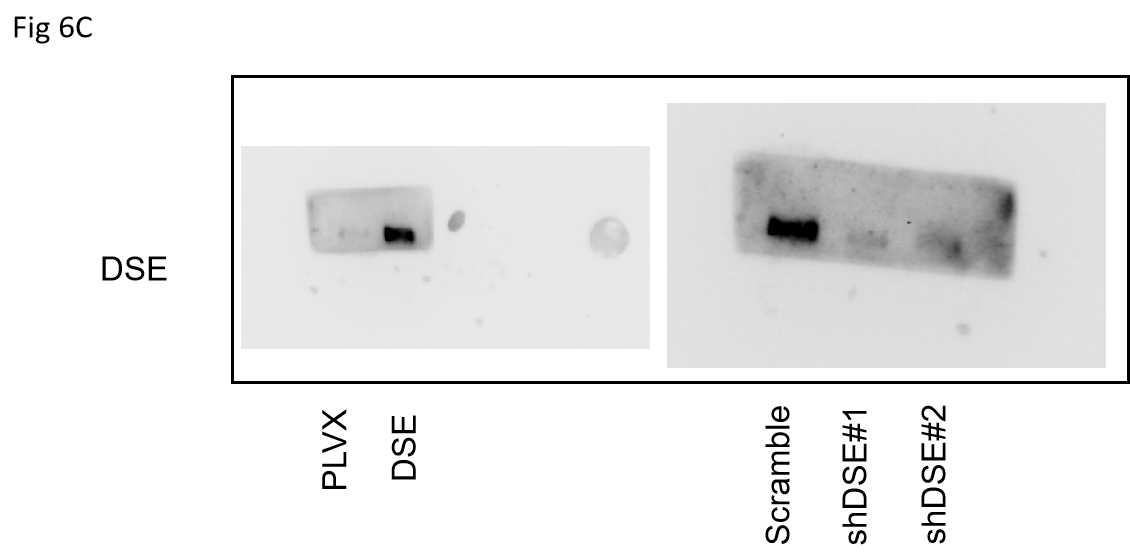


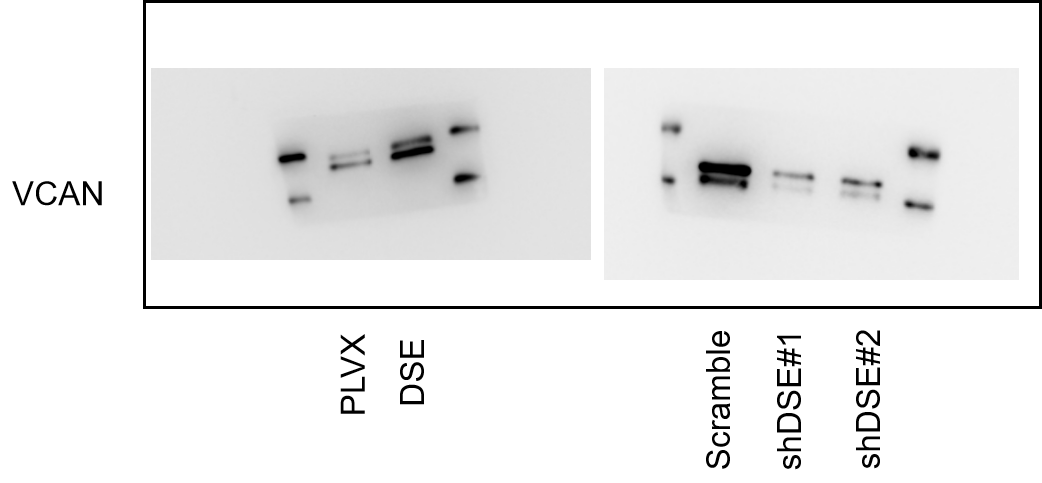


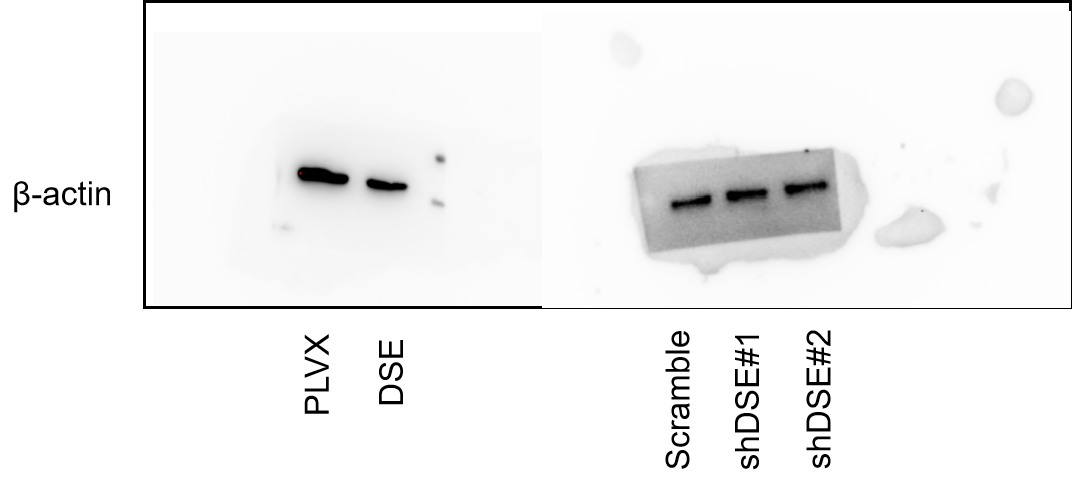


Fig 6E


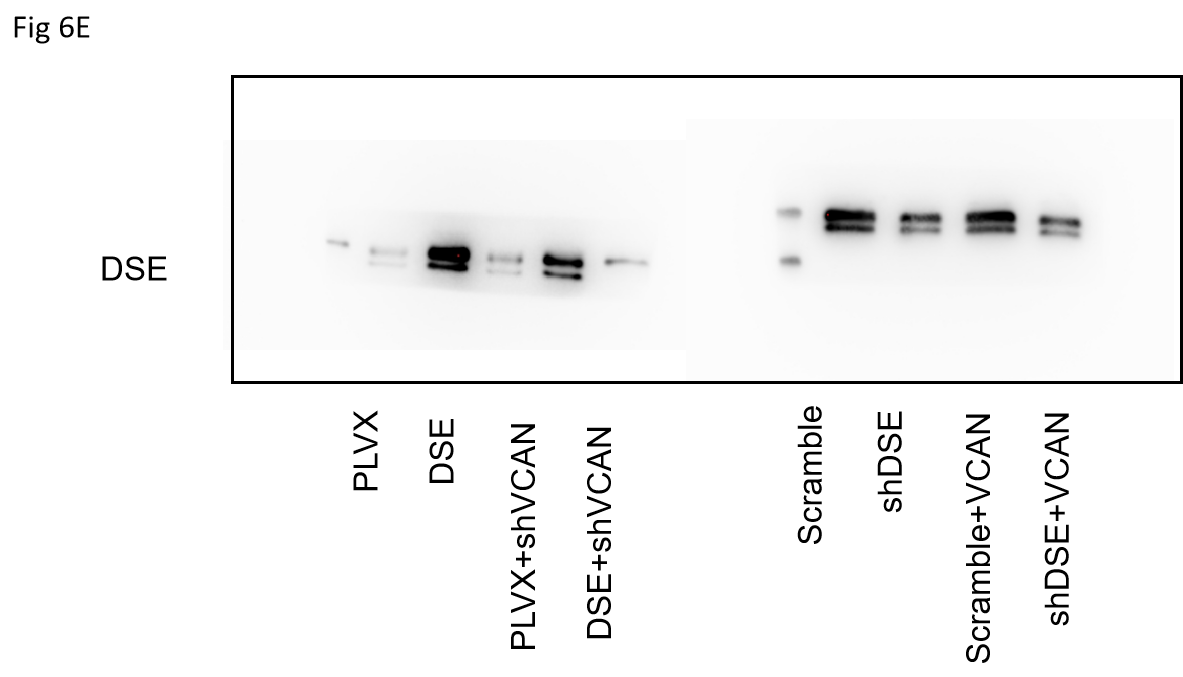


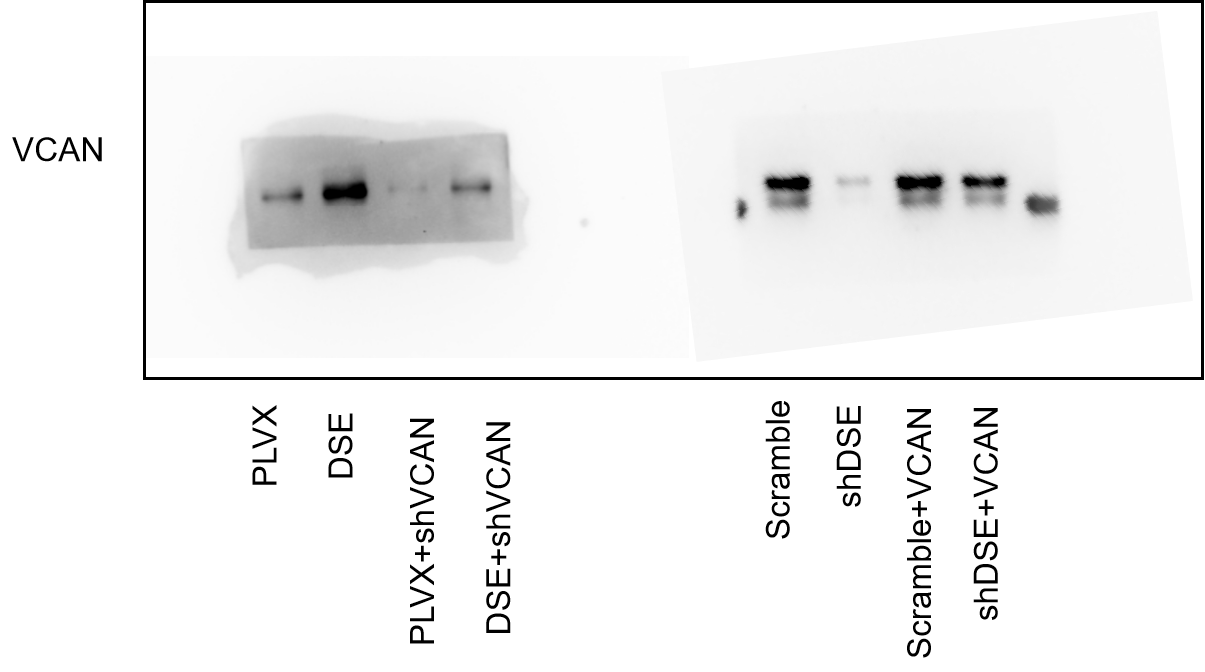

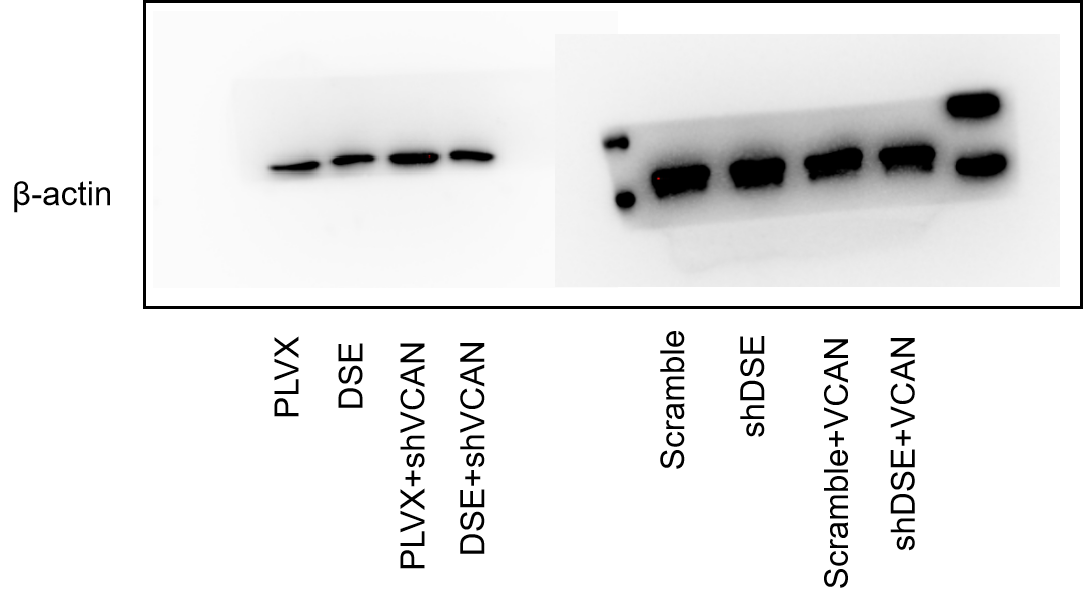

Supplement: Supplementary file 1 — Original Data File [file 41420_2023_1676_MOESM1_ESM.docx]
