# Supplementary material for: DSE inhibits melanoma progression by regulating tumor immune cell infiltration and VCAN
Source: Cell Death Discov. 2023 Oct 13;9:373. doi: 10.1038/s41420-023-01676-8 (PMC10576081; doi:10.1038/s41420-023-01676-8)
Supplement: Supplementary file 2 — Supplementary Material [file 41420_2023_1676_MOESM2_ESM.docx]

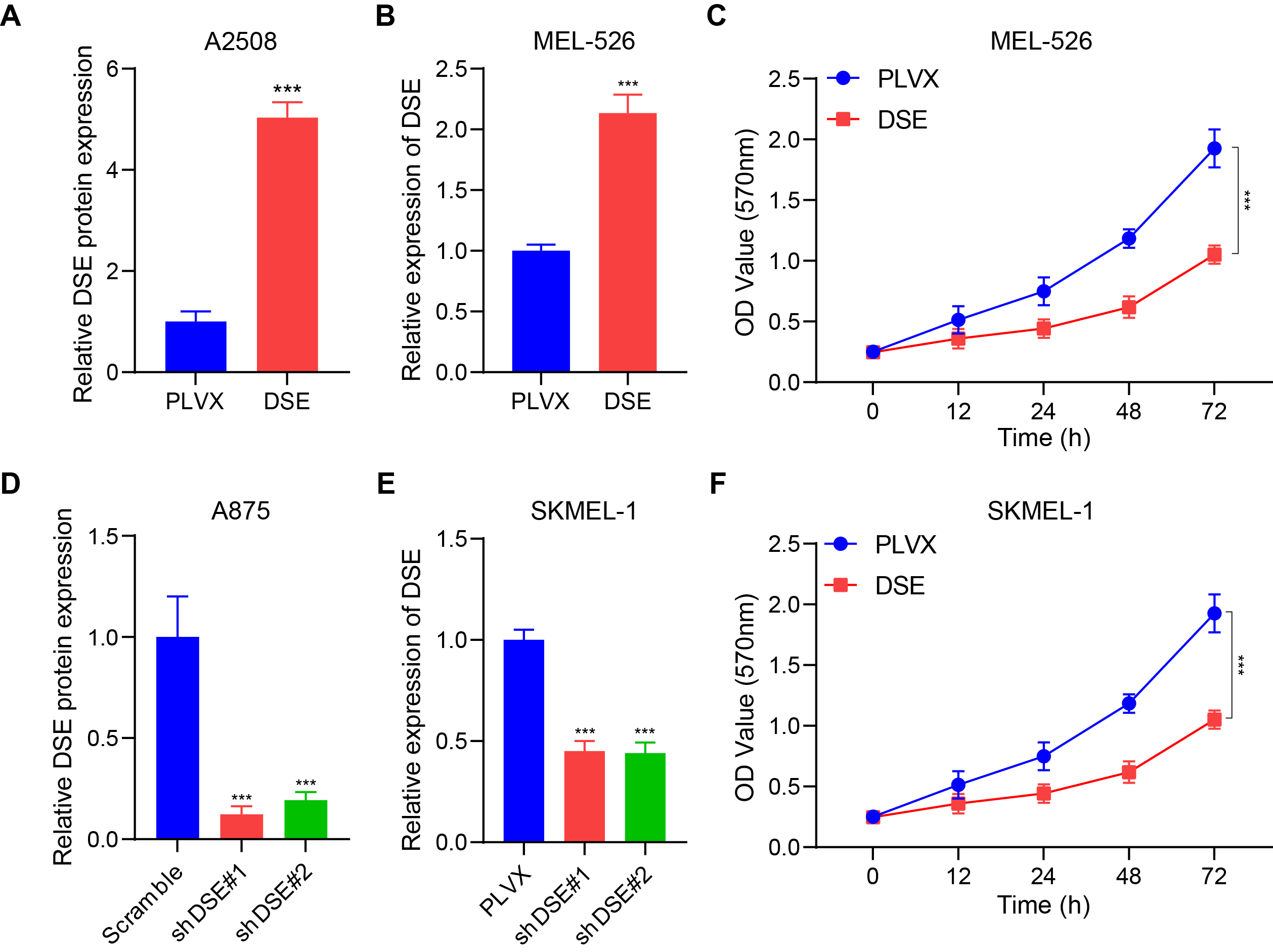


**Supplementary Figure 1. DSE inhibits proliferation, invasion and migration of melanoma cells. A)** A2508 cells were infected with lentivirus (PLVE) and DSE, respectively, and the expression levels of DSE protein in cells were analyzed by western blotting; **B-C)** CCK8 assay to analysis cell viability of MEL-526 cells stably overexpressing DSE (mean ± s.e.m. ****P* < 0.001); **D)** Knockdown of DSE in A875 cells and Western blot analysis of DSE protein expression levels in those cells; **E-F)** CCK8 assay was used to analyze the cell viability of DSE knockout SKMEL-1 cells (mean ± s.e.m. ****P* < 0.001).


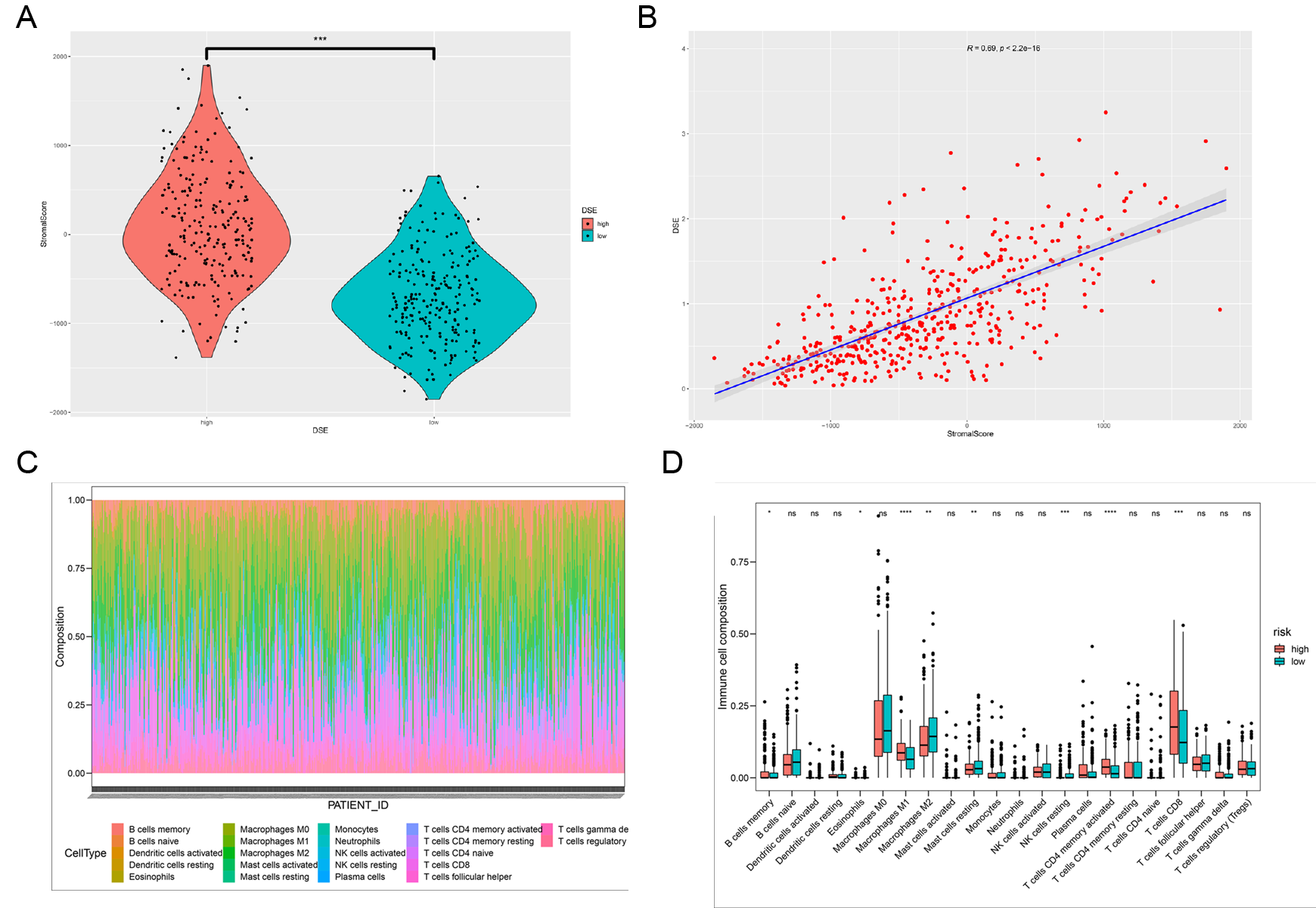


**Supplementary Figure 2. ESTIMATE and CIBERSORT analysis of immune infiltration in melanoma patients from the TCGA database. A, B)** ESTIMATE analysis of DSE correlation with stromal score in tumor tissue. **C)** CIBERSORT analysis of immune infiltration in melanoma patients in the TCGA database; **D)** Correlation analysis of DSE expression levels and immune infiltrating cells.


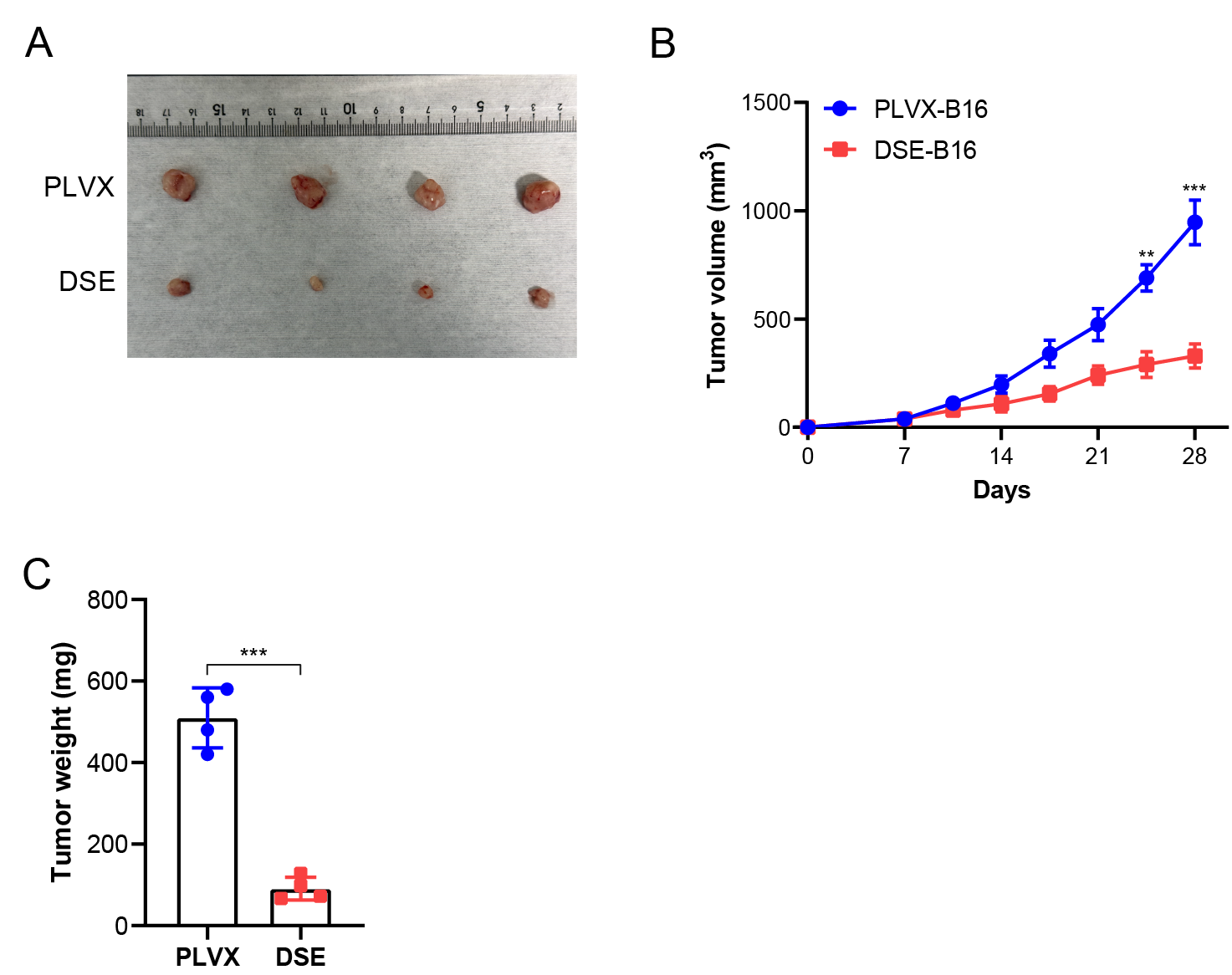


**Supplementary Figure 3. B16 cells stably overexpressing DSE were injected into C57 mice to construct a melanoma tumor model (related to Figure 4C-E)**. A) Morphology of tumor; B) Growth curve of tumor (n = 4 mice, mean ± s.e.m. ***P* < 0.01, ****P* < 0.001); C) Weight of tumor (n = 4 mice, mean ± s.e.m. ****P* < 0.001).


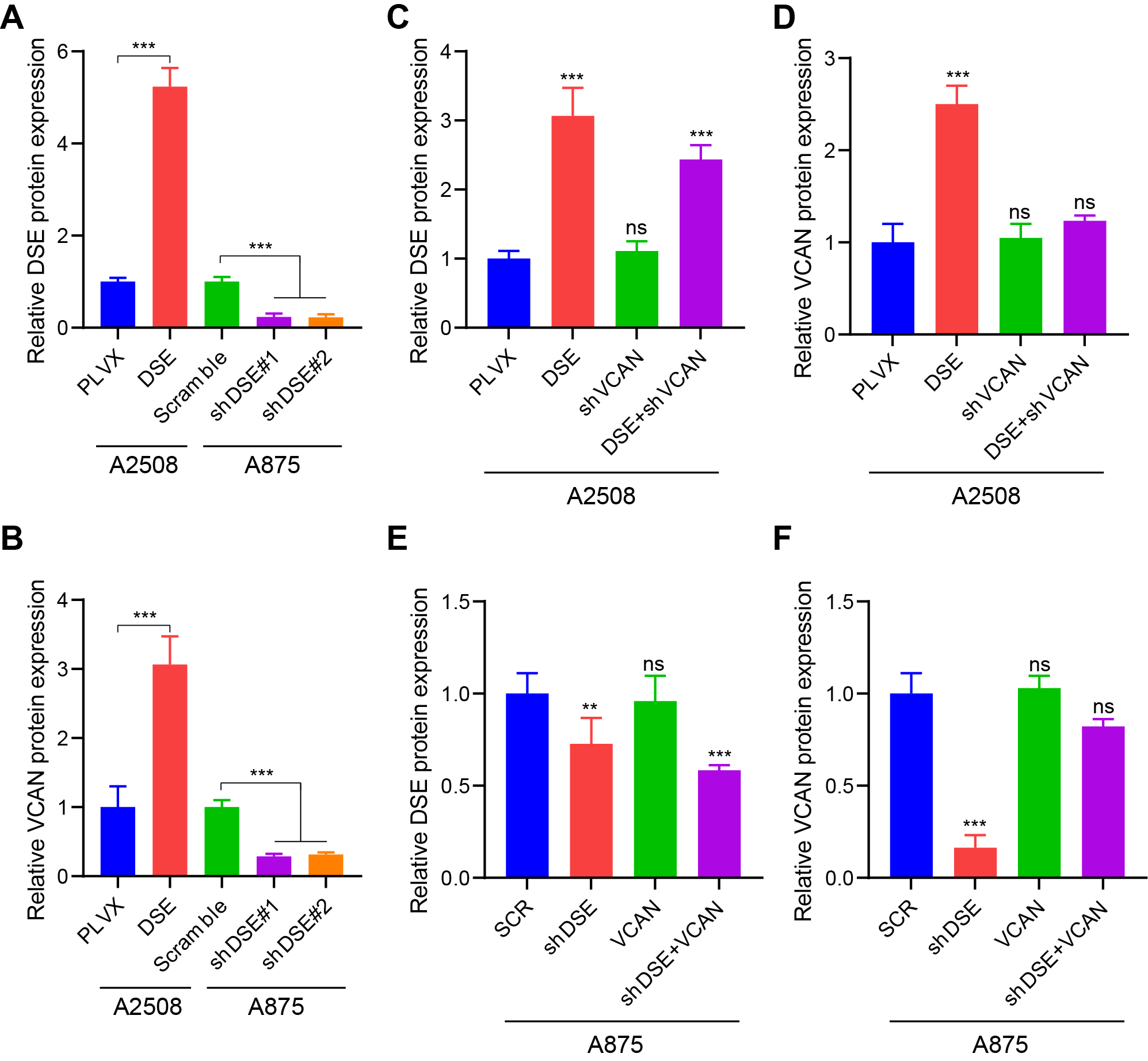


**Supplementary Figure 4. DSE regulates the expression of VCAN.** The DSE and/or VCAN protein expression in DSE-overexpressing or knockdown cells level (mean ± s.e.m. ***P* < 0.01, ****P* < 0.001)
